# RESVEGA, a Nutraceutical Omega-3/Resveratrol Supplementation, Reduces Angiogenesis in a Preclinical Mouse Model of Choroidal Neovascularization

**DOI:** 10.3390/ijms222011023

**Published:** 2021-10-13

**Authors:** Flavie Courtaut, Virginie Aires, Niyazi Acar, Lionel Bretillon, Ida Chiara Guerrera, Cerina Chhuon, Jean-Paul Pais de Barros, Céline Olmiere, Dominique Delmas

**Affiliations:** 1 Université de Bourgogne Franche-Comté, 21000 Dijon, France; flavie.courtaut@gmail.com (F.C.); virginie.aires02@u-bourgogne.fr (V.A.); niyazi.acar@inrae.fr (N.A.); Lionel.Bretillon@u-bourgogne.fr (L.B.); jppais@u-bourgogne.fr (J.-P.P.d.B.); 2 INSERM Research Center U1231—Cancer and Adaptive Immune Response Team, Bioactive Molecules and Health Research Group, 21000 Dijon, France; 3 Centre des Sciences du Goût et de l’Alimentation, AgroSup Dijon, CNRS, INRAE, Eye and Nutrition Research Group, 21000 Dijon, France; 4 Proteomics Platform Necker, Université de Paris—Structure Fédérative de Recherche Necker, INSERM US24/CNRS UMS3633, 75015 Paris, France; chiara.guerrera@inserm.fr (I.C.G.); cerina.chhuon@inserm.fr (C.C.); 5 INSERM Research Center U1231-Lipidomic Analytical Platform, 21000 Dijon, France; 6 Laboratoires Thea, 12 Rue Louis-Blériot, 63000 Clermont-Ferrand, France; celine.olmiere@theaopeninnovation.com; 7 Centre Anticancéreux Georges François Leclerc, 21000 Dijon, France

**Keywords:** AMD, angiogenesis, ocular diseases, proteomic, omega-3 fatty acids, resveratrol

## Abstract

Age-related macular degeneration (AMD) is an eye disease that is characterized by damage to the central part of the retina, the macula, and that affects millions of people worldwide. At an advanced stage, a blind spot grows in the center of vision, severely handicapping patients with this degenerative condition. Despite therapeutic advances thanks to the use of anti-VEGF, many resistance mechanisms have been found to accentuate the visual deficit. In the present study, we explored whether supplementation with Resvega^®^, a nutraceutical formulation composed of omega-3 fatty acids and resveratrol, a well-known polyphenol in grapes, was able to counteract laser-induced choroidal neovascularization (CNV) in mice. We highlight that Resvega^®^ significantly reduced CNV in mice compared with supplementations containing omega-3 or resveratrol alone. Moreover, a proteomic approach confirmed that Resvega^®^ could counteract the progression of AMD through a pleiotropic effect targeting key regulators of neoangiogenesis in retina cells in vivo. These events were associated with an accumulation of resveratrol metabolites within the retina. Therefore, a supplementation of omega-3/resveratrol could improve the management or slow the progression of AMD in patients with this condition.

## 1. Introduction

Age-related macular degeneration (AMD) is the main cause of deteriorated vision in adults aged 50 or older in developed countries, and it remains a major public health problem with an obvious impact on quality of life [1]. There are two forms of AMD. The first is the atrophic or “advanced dry” form, which is characterized by a narrowing of the retina and accumulation of proteins. It results in a progressive loss (over 5 to 10 years) of central vision in one eye. Details are blurry, but color perception and vision remain intact. The second is the wet form, which is characterized by the development of new blood vessels in the macula. Vision loss in this form of AMD is much faster (a few days to a few weeks without treatment). There is no treatment for the dry form of AMD, but monitoring is essential to limit complications (progression to the wet form). For the wet form of the disease, there are several treatments targeting the blood vessels: anti-angiogenic or anti-VEGF treatments (i.e., ranibizumab, bevacizumab, aflibercept, etc.) [2]. The purpose of these drugs is to stabilize the disease by preventing the development of new blood vessels in the macula. In addition to these pharmacological strategies, for which resistance sometimes appears, numerous studies have shown the influence of nutrition on the occurrence of this disease and its progression [3,4]. Nutrient supplementation could play a role, specifically for fatty acids, which is a category of lipids that play a fundamental role in cell structure and energy storage. Polyunsaturated fatty acids (PUFAs), especially omega 3 (fatty fish such as salmon, tuna, mackerel), as well as fruits and vegetables (rich in zeaxanthin and lutein) appear to be beneficial. In addition, antioxidants can contribute by scavenging free radicals, which are toxic compounds resulting from the “respiration” of cells. Vitamins C and E, polyphenols, and certain minerals (zinc, selenium) may reduce the risk of developing early and late forms of AMD. The Age Related Eye Disease Study 1 (AREDS-1), a multicenter randomized controlled clinical trial, demonstrated that oral nutritional supplementation with a combination of vitamin C, vitamin E, β-carotene, zinc oxide, and cupric oxide in patients with intermediate or advanced AMD in one eye had a 25% relative risk reduction over 5 years of developing advanced AMD. The risk of vision loss of three or more lines was also reduced by 19% with this supplementation regime [3]. Moreover, a French study (NAT1, Nutritional AMD Treatment 1) showed that lesions due to AMD were stabilized in patients supplemented with a PUFA—namely, docosahexaenoic acid (DHA) [4]. More recently, studies have shown that polyphenols could also prevent or improve vision in patients with ocular diseases and especially AMD [5]. For example, one randomized double-blind clinical trial including 72 patients showed that supplementation with *trans*-resveratrol (RSV) lowered VEGF levels in peritoneal effluent [6], thereby supporting the notion that RSV could be effective at reducing neovascularization in patients. Another clinical trial performed in the USA in octogenarians also showed that oral administration of Longevinex^®^, which is a combination of RSV with quercetin, ferulic acid together with vitamin D3, and a cooper/iron/calcium binding molecule called IP6 (inositol hexaphosphate), could improve retinal structure and visual function [7] and reduce neovascularization [8]. Very recently, Resvega^®^ (RSG) was developed using the AREDS recommendations in terms of fatty acid and antioxidant composition, to which a quantity of resveratrol was added. In a previous study, we have shown that this RSG nutraceutical formulation composed of both omega-3 fatty acids (ω3) and RSV can reduce vascular endothelial growth factor (VEGF) through a disruption of its receptor activation, VEGF-R2. Indeed, RSG relocalized VEGF-R2 into lipid rafts, favoring its association to caveolin-1 and inducing an inhibition of the signaling kinase pathway, which in AMD leads to an overexpression of VEGF production in retinal cells mimicking AMD. Other authors have also confirmed an interest in this formulation for various molecular processes such as inflammation [9], oxidative stress [10], and autophagy [11] in retinal cell models.

The ability of RSG supplementation to modulate neoangiogenesis in vivo still needs to be explored. In the present study, we investigated the effects of three nutraceuticals (RSG containing both RSV and ω3 fatty acids (EPA, eicosapentaenoic acid; DHA, docosahexaenoic acid); Nutrof^®^ (NUT), a nutraceutical formulation containing ω3 fatty acids without RSV; and RSV alone) on angiogenesis in a preclinical mouse model of choroidal neovascularization (CNV) induced by laser photocoagulation. We demonstrate that there was a significant reduction in the development of CNV in the RSG and RSV supplementation groups in comparison with the control and NUT supplementation groups. In addition, a vast proteomic analysis of the retina from mouse models confirmed the effect of RSG on a range of proteins involved in the control of neoangiogenesis.

## 2. Results

### 2.1. Supplementation with ω-3 Fatty Acids/RSV Combination Reduces Laser-Induced CNV in Mice

**Figure 1:**
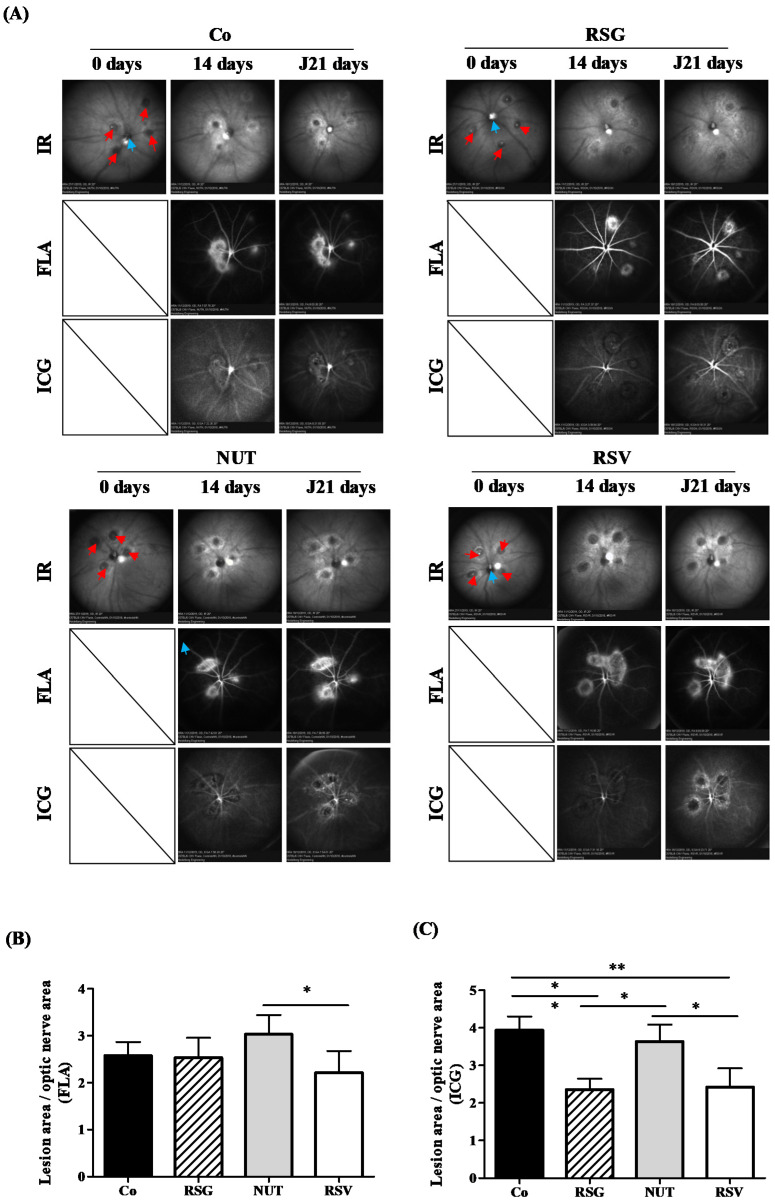
RSG decreases laser-induced CNV in mice. (**A**) Fundus images of mice that were either untreated (Co) or supplemented with RSG (12 µM), NUT (12 µM), or RSV (20 µM) for 14 days prior to CNV induction. Infrared (IR) pictures show the four laser impacts at day 0 (red arrows) as well the optic nerve head positioned at the center of the image (blue arrows). Vascular lesions around laser spots are visible at 14 and 21 days following CNV induction on fluorescein (FLA) and indocyanin green (ICG) angiographies at inner retinal and choroidal levels, respectively. (**B**) Quantitative analysis of vascular lesion areas in the inner retina did not reveal significant differences between animals supplemented with RSG, NUT, and RSV when compared with untreated mice, whereas (**C**) a focused evaluation at the level of choroid showed significantly lower development of CNV in animals supplemented with RSG and RSV. Data are presented as means ± SEM. *p* values were determined by a one-way ANOVA followed by a Mann–Whitney test. * *p* < 0.05 and ** *p* < 0.01.

First, to test the potential antiangiogenic effects of the nutraceutical formulations, we used a laser-induced choroidal neovascularization (CNV) mouse model. This model is usually used to provide preclinical evidence to support the clinical evaluation of anti-VEGF drugs for neovascular eye diseases such as AMD and to evaluate new therapies for these conditions [12,13]. Thus, after validation by the ethics committee of the university, we used this “gold standard” preclinical method, which is very well codified and documented in the literature [14]. Briefly, in this model, targeted laser injury to the retinal pigment epithelium and Bruch’s membrane induces pathological angiogenesis in the basement membrane of the retina, mimicking the hallmark pathology observed in neovascular AMD. First, to determine whether nutraceutical supplementation could protect from the occurrence of AMD or prevent the progression of CNV, we supplemented C57BL/6 mice for 14 days with the different nutraceuticals. We previously adapted the doses used in human nutrition to mice—namely, 6 mg/kg [15]—so as to have the most faithful transposition in this model of CNV induced by laser photocoagulation. At 14 days, the four groups of mice (Co, control; RSG, ω-3/RSV combination; NUT; and RSV) were subjected to four laser applications on one eye and then continued to receive their daily dose of nutraceuticals for 21 days. On days 14 and 21 following the laser treatments, mice were subjected to angiographic examinations in order to determine whether the nutraceuticals were able to prevent the wet AMD phenotype by inhibiting CNV (Figure 1). In vivo fluorescein angiography (FLA), which can be used to dynamically visualize inner retinal vessels, revealed that the different treatments had no significant influence on vessel development within the inner retina (Figure 1A,B). Very interestingly, indocyanine green angiography (ICG), which facilitates a deeper penetration and subsequently a direct visualization of the choroidal vessels and CNV, highlighted a significant reduction in CNV development in the animals supplemented with RSG compared with the control or other treatments (NUT and RSV) (Figure 1A,C). The combination of ω-3/RSV acted to drastically reduce the surface invaded by the new vessels compared with the control treatment or treatment with ω-3 alone (Figure 1A). These in vivo results show for the first time that RSG could reduce CNV involved in the growth of new blood vessels after laser induction.

### 2.2. Proteomic Analysis and Targets of Combined ω-3/RSV in a CNV Mouse Model

**Figure 2:**
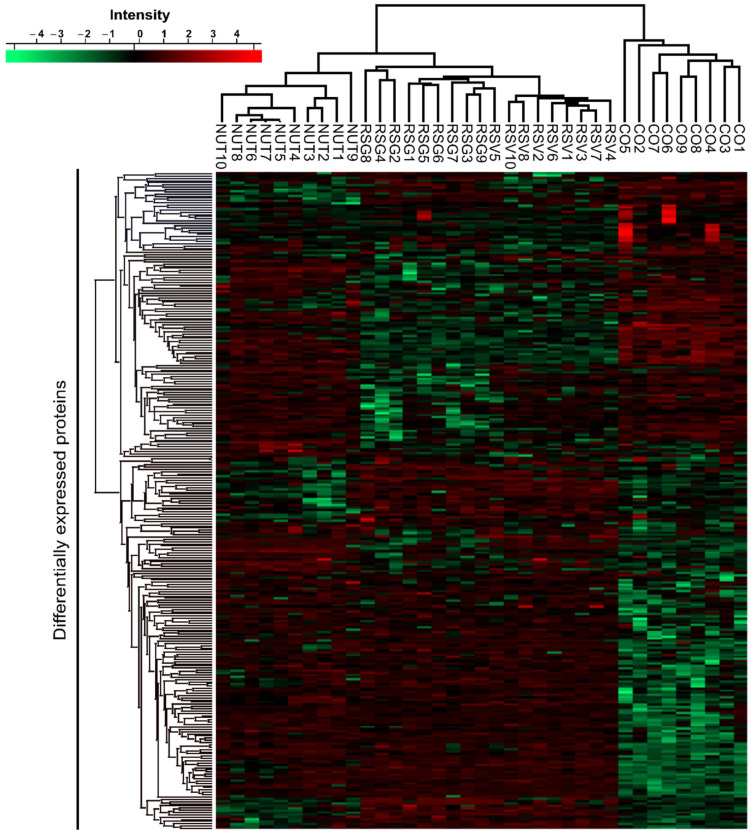
Differentially expressed proteins (DEPs) in lasered retinas of mice supplemented with RSG, NUT, and RSV nutraceuticals. Heatmap of DEPs. Hierarchical clustering was performed on logarithmized LFQ intensities after z-score normalization of the data, using Euclidean distances (ANOVA test, FDR < 0.05, S0 = 1). Red indicates a high expression level and green a low expression level.

In order to support the angiographic results, we performed a global proteome analysis on lasered retinas from the groups of mice (9–10 retinas per group). Of the 3570 proteins identified in the retina samples, 291 were differentially expressed across mice diet groups (Figure 2). Differentially expressed proteins made it possible to distinguish the proteome of mice on the control diet from the proteome of mice on the other diets. We also observed that the proteome of the retinas from mice fed with RSV and RSG were more similar to each other than to the NUT diet (see dendrogram issues from hierarchical clustering of the samples on top of the heatmap). To begin, using ANOVA tests, we carried out pathway and process enrichment analyses on all differentially expressed proteins (DEPs) using the Metascape online tool (http://metascape.org, (accessed on 7 July 2021)). The Kyoto Encyclopedia of Genes and Genomes (KEGG) pathways, Gene Ontology (GO) biological processes, GO cellular component, GO molecular function, Reactome Gene Sets, and CORUM were used as ontology sources. All genes in the mouse genome were used as the enrichment background. Statistically enriched terms (minimum count of three) were filtered based on the calculated accumulative hypergeometric *p*-values (<0.01), corrected for multiple testing and enrichment factor (ratio between observed count and count expected by chance) >1.5. The remaining significant terms were then hierarchically clustered into a tree based on kappa—statistical similarities among membership similarities. Then, the 0.3 kappa score was applied as the threshold to cast the tree into term clusters.

**Figure 3:**
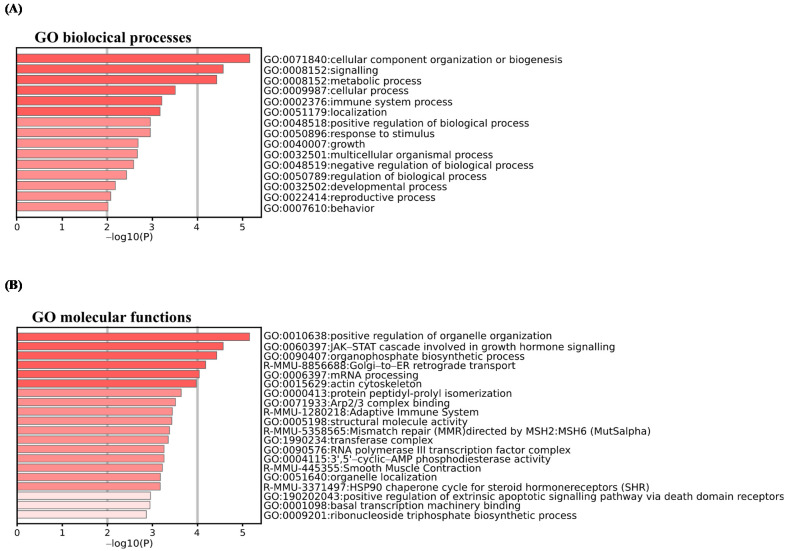
Pathway and process enrichment analysis using Metascape database for DEPs. Enriched pathways and processes were identified using Metascape database (http://metascape.org, (accessed on 7 July 2021)) for all DEPs from ANOVA test and with Kyoto Encyclopedia of Genes and Genomes (KEGG) pathways, Gene Ontology (GO) biological processes, GO cellular component, GO molecular function, Reactome Gene Sets, and CORUM as ontology sources. Top enrichment biological processes (**A**) and molecular functions (**B**) term clusters, colored by −log_10_ (*p*-value). Threshold: 0.3 kappa score; similarity score > 0.3. The darker the color is, the more significant the term. Significant terms were then hierarchically clustered into a tree based on kappa—statistical similarities among membership similarities. Then, the 0.3 kappa score was applied as the threshold to cast the tree into term clusters. More specifically, the most statistically significant term within a cluster was selected for cluster annotation. The first 20 significant most representative term clusters are shown.

More specifically, the most statistically significant term within a cluster was selected for cluster annotation. The top 20 clusters and most representative term clusters and their corresponding −log_10_ (*p*-values) are displayed in Figure 3A,B. We found that DEPs were associated with GO biological processes related to signaling, cellular and immune system, and positive or negative regulation (Figure 3A). The associated GO molecular functions notably included positive regulation of organelles organization, Janus kinase/signal transducers and activators of the transcription (JAK-STAT) cascade involved in growth hormone signaling pathways, the adaptive immune system, and positive regulation of extrinsic signaling pathways via death domain receptors (Figure 3B).

### 2.3. Resvega^®^ Up-Regulates Gene Ontology Clusters Associated with Negative Regulation of Epithelial Cell Migration

**Figure 4:**
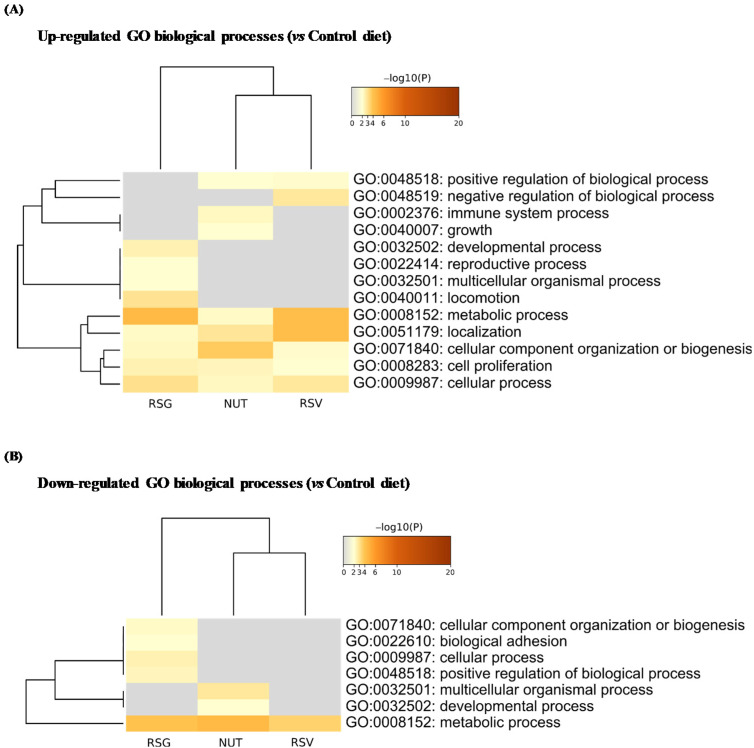
Enrichment analysis of Gene Ontology (GO) biological processes in differentially up- and down-regulated proteins from lasered mice retina. Based on ANOVA results, *t*-tests with Welch correction were further applied to identify significantly up- and down-regulated proteins in RSG, NUT, and RSV as compared with the control diet group (Co). Metascape database was then used to identify Gene Ontology (GO) enriched biological processes in up- (**A**) and down-regulated DEPs (**B**). Heatmaps show the top enrichment biological processes in up-regulated (**A**) and down-regulated DEP (**B**) term clusters, colored by −log_10_ (*p*-value). Threshold: 0.3 kappa score; similarity score > 0.3. The darker the orange is, the more significant the term. Gray color indicates a lack of significance.

**Figure 5:**
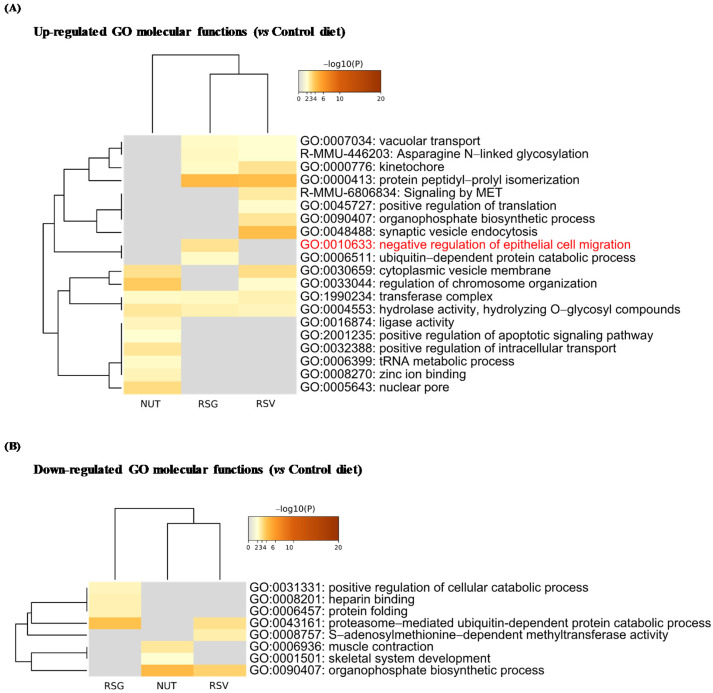
Enrichment analysis of Gene Ontology (GO) molecular functions in differentially up- and down-regulated proteins from lasered mice retina. Based on ANOVA results, *t*-tests with Welch correction were further applied to identify significantly up- and down-regulated proteins in RSG, NUT, and RSV groups compared with the control diet group (Co). Metascape database was then used to identify Gene Ontology (GO) enriched molecular functions in up- (**A**) and down-regulated DEPs (**B**). Heatmaps show the top enriched molecular functions in up-regulated (**A**) and down-regulated DEP (**B**) term clusters, colored by −log_10_ (*p*-value). Threshold: 0.3 kappa score; similarity score > 0.3. The darker the orange is, the more significant the term. Gray color indicates a lack of significance.

We next identified significantly up- and down-regulated proteins in the RSG, NUT, and RSV groups compared with the control group (Co) and used the multi-gene-list meta-analysis mode of Metascape to highlight overlapping or selective enriched clusters and pathways across experimental groups. More specifically, significant terms were hierarchically clustered into GO groups and were visualized as a heatmap (color scale represents −log_10_ (*p*-values); gray color indicates a lack of significance). Dendrograms show the closeness of enriched term clusters (one per row) and the closeness of input DEPs lists across experimental groups (one per column) (Figure 4 and Figure 5).

As for biological processes, the up-regulated DEPs were significantly enriched in metabolic process, localization, cellular component organization, cell proliferation, and cellular process in the RSG, RSV, and NUT experimental groups (Figure 4A). However, we found selective enrichment in GO components in experimental groups—for instance, the specific enrichment of GO: 0002376 (immune system process) and GO: 0040007 (growth) in the retinas from NUT-supplemented mice (Figure 4A). Interestingly, for molecular functions, we found specific enrichment in GO: 0010633 negative regulation of epithelial cell migration cluster (5.36% of enrichment, −log_10_ (*p*-value) = 3.00) in the retinas from RSG-supplemented mice (Figure 5A).

**Figure 6:**
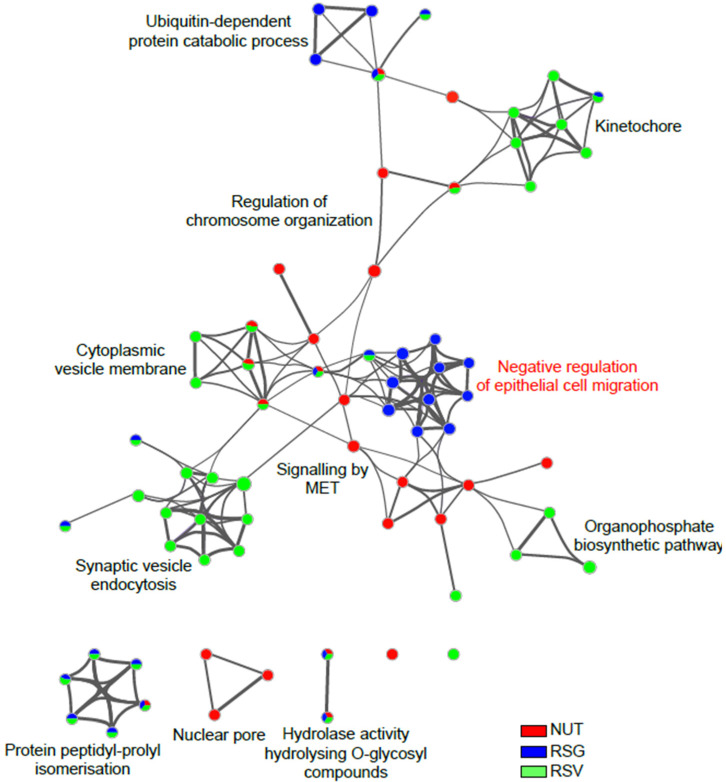
Network visualization of Gene Ontology (GO) molecular function clusters in differentially down-regulated proteins from lasered mice retina. Enriched terms in significantly down-regulated proteins in RSG, NUT, and RSV groups compared with the control diet group (Co) were selected and rendered as a network plot to further determine the relationship among terms, where terms with similarity of >0.3 were connected by edges. The network of enriched sets colored according to experimental group identity was visualized with Cytoscape (v3.1.2) with “force-directed” layout and with bundled edges. The size of a node is proportional to the number of input DEPs that fall into the term and slices within each node represents the number of proteins under the term that originate from each experimental diet group.

Representative terms from the full cluster were further converted into a network layout and visualized with Cytoscape (v3.1.2). More specifically, the top 20 clusters were sampled and selected (10 best scoring terms within each cluster with the lowest *p*-value), represented as a circle node, and then all term pairs with kappa similarity above 0.3 were connected by edges. The size of the node was proportional to the number of input DEPs that fell into that term, and was colored according to its experimental group identity. The size of the slices within each node represent the percentage of proteins under the term that originated from each experimental diet group (Figure 6). Negative regulation of the epithelial cell migration cluster, which is specifically enriched in retina from RSG supplemented mice group, included GO terms related to: negative regulation of cell migration (GO: 0010633, −log_10_ (*p*-value) = 2.99; GO: 0030336, −log_10_ (*p*-value) = 2.15), vasculature development (GO: 0001944, −log_10_ (*p*-value) = 2.29), blood vessel development (GO: 0001568, −log_10_ (*p*-value) = 2.41), angiogenesis (GO: 0001525, −log_10_ (*p*-value) = 2.51), blood vessel morphogenesis (GO: 0001525, −log_10_ (*p*-value) = 2.10) and negative regulation of epithelial cell proliferation (GO: 0001935, −log_10_ (*p*-value) = 2.11; GO: 0050673, −log_10_ (*p*-value) = 2.20; GO: 0050680, −log_10_ (*p*-value) = 2.12). The identity of the hit proteins that fell in that cluster were phosphatase and TENsin homolog (PTEN), pigment epithelium-derived factor (SERPINF1), programmed cell death protein 10 (PDCD10), ER membrane protein complex subunit 10 (EMC10), signal transducer and activator of transcription 3 (STAT3), E3 ubiquitin-protein ligase Itchy homolog (Itch), sushi repeat-containing protein SRPX (SRPX), beclin-1 (BECN1), transmembrane emp24 domain-containing protein 2 (TEMD2), nuclear pore complex protein Nup107 (NUP107), translation initiation factor eIF-2B subunit epsilon (Eif2b5), and Bcl2-associated agonist of cell death (Bad).

Overall, these data highlight the potential of nutraceuticals composed of polyphenols and ω-3 fatty acids to counteract the progression of AMD through a pleiotropic effect targeting key regulators of neoangiogenesis in retina cells in vivo.

### 2.4. RSV Metabolites from RSG Accumulate in the Retina and Posterior Pole

**Figure 7:**
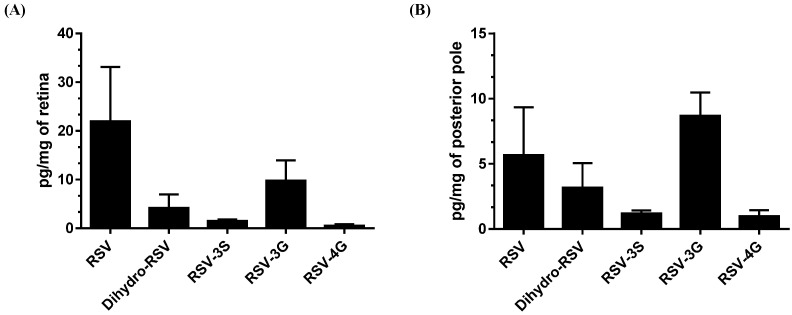
Resveratrol aglycone and resveratrol metabolites accumulate in retinal tissue of mice. (**A**) Analysis of RSV metabolites and RSV aglycone in retina lysates and (**B**) posterior pole in mice (*n* = 5) treated with RSG (12 µM) for 21 days.

These results led us to explore whether the anti-angiogenic effects observed were correlated with the presence of RSV metabolites in the structures of eye. In the context of colorectal cancer, various metabolites were found to be accumulated in tumor tissues after RSV treatment [16]. In addition, we have previously shown that these RSV metabolites can exert a potent biological effect [17,18]. In a parallel experiment, we supplemented C57BL/6 mice for 21 days with daily 6 mg/kg of RSG without laser photocoagulation. At 21 days, we isolated the retinas and posterior pole of the eyes and analyzed the levels of resveratrol and its main metabolites by high-performance liquid chromatography (HPLC) to determine whether they were found in these structures. Very interestingly, we detected metabolites of RSV and the aglycone molecule in the retina and the posterior pole (Figure 7). Indeed, we identified two resveratrol metabolites in the retina lysate—namely, dihydro resveratrol (Dihydro-RSV) and resveratrol-3-O-glucuronide (RSV-3G) at concentrations of 9 pg/mg and 13 pg/mg of retina, and very low level for resveratrol-3-O-sulfate (RSV-3S) with 1.5 pg/mg (Figure 7A). Concerning the posterior poles, we found the aglycone molecule from resveratrol at a concentration of 11.34 pg/mg, as well as several metabolites (dihydro-RSV at 6.36 pg/mg, RSV-3S at 1.2 pg/mg, RSV-3G at 8.7 pg/mg, and resveratrol-4-O-glucuronide (RSV-4G) at 0.98 pg/mg) (Figure 7B). These preliminary results should be completed by future studies assessing the bioavailability of RSV and its pharmacokinetics. However, these findings suggest that the metabolites of resveratrol could play a role in the biological effects observed, as previously demonstrated in the context of colon cancer or myopathy [17,18].

## 3. Discussion

AMD is the leading cause of reduced visual acuity in patients over 65 years of age, and, despite the development of new anti-VEGF antibodies to counteract neovascularization (i.e., ranibizumab, bevacizumab, aflibercept, etc.) [2], treatment failure still occurs due to side effects and resistance. Herein, through the use of potential anti-angiogenic nutraceuticals, we describe for the first time the potential of combined ω-3 fatty acids and RSV (RSG) to counteract choroidal neovascularization (CNV) through the down modulation of key proteins involved in angiogenesis, as revealed by a proteomic analysis.

The pathological process of AMD leads to progressive destruction of the neurosensory macular area, involving the retinal pigment epithelium, Bruch’s membrane, and the choroid. Different stages of the disease have been described. The early stage is an age-related maculopathy (MLA) characterized by drusen and/or alteration of the pigment epithelium. It may cause a moderate decrease in vision or a distortion of the images (metamorphopsia). The more advanced stages include atrophic or dry AMD, which is the most common and less severe form. It only leads to symptoms when the patches of atrophy come together and touch the center of the macula. There is then a loss of vision. On the other hand, the second advanced form, exudative or wet AMD, is linked to a proliferation of abnormal blood vessels under the retina, starting from the choroid. These so-called choroidal neovessels are fragile and can cause retinal edema or hemorrhage. The progression is often rapid, leading to a loss of central vision within a few weeks or months without treatment. In this context, pro-angiogenic VEGF-A had been shown to be involved in the development of CNV [19]. Based on their use in treatments for various metastatic cancers, VEGF inhibitors have revolutionized the care of vasoproliferative ophthalmologic disease. However, these therapies can have side effects, and resistance has been observed. Subsequently, researchers are looking for alternatives to improve the management of this serious disease. For many years, various studies have sought effective approaches to prevent disease occurrence and progression. In doing so, the Age Related Eye Disease Study 1 (AREDS-1), a multicenter randomized controlled clinical trial, demonstrated that patients with intermediate or advanced AMD in one eye given oral nutritional supplementation of a combination of vitamin C (500 mg), vitamin E (400 UI), β-carotene (15 mg), zinc oxide (80 mg), and cupric oxide (2 mg) had a relative risk reduction of 25% of developing advanced AMD in the eye over 5 years. The risk of vision loss of three or more lines was also reduced by 19% with this treatment. Several other epidemiological studies demonstrated that carotenoid intake reduced the risk of advanced AMD and that a lutein- and zeaxanthin-based diet might protect against intermediate AMD in female patients. Therefore, the objective of the second AREDS (AREDS-2) was to determine whether the addition of lutein/zeaxanthin and omega-3 fatty acids (docosahexaenoic (DHA) and eicosapentaenoic acids (EPA)) would further reduce progression to late-stage AMD. Unfortunately, the addition of lutein + zeaxanthin, DHA + EPA, or lutein + zeaxanthin and DHA + EPA to the complete AREDS formulation did not further reduce the risk of progression to advanced AMD [3,20]. However, because of the potential increased incidence of lung cancer in former smokers, lutein + zeaxanthin could be an appropriate carotenoid substitute in the AREDS formulation [20]. Nevertheless, other studies have shown that long-chain polyunsaturated fatty acids could protect against AMD [4]. Indeed, a meta-analysis suggested that ω-3 fatty acids (such as DHA), which are essential dietary compounds found in fish such as salmon or tuna, could reduce the risk of both early and late AMD [21]. Similarly, other natural compounds such as polyphenols could act on the different molecular steps of AMD through antioxidant, anti-inflammatory, and anti-angiogenic pleiotropic effects [22,23,24]. Various nutraceutical formulations including RSV have been developed, and the first clinical tests have shown various beneficial effects. For example, a randomized clinical study showed that the RSV group (taking pills containing 100 mg of RSV) increased choroidal thickness (by EDI-OCT) one hour after RSV administration compared with baseline measurements [25]. Moreover, in three cases of patients taking 100 mg doses of RSV for 2.5 years, Ritcher et al. reported broad bilateral improvements in retinal and choroid structure and function, visual acuity, contrast sensitivity, and glare recovery over a long time period, contrary to what might be expected due to aging and the natural progression of pathophysiology [7]. Very interestingly, these beneficial effects were obtained without side effects since the doses used in these studies were much lower than those for which side effects have been observed, in particular from 2.5 g per day up to 5 g per day [16,26]. Thus, in order to combine the AREDS recommendations for omega-3 fatty acid supplementation with the previously described beneficial effects of RSV, a formulation called Resvega^®^ was developed, containing mainly eicosapentaenoic acid, docosahexaenoic acid, and resveratrol. While it has no visible action on vessel development within the inner retina, RSG significantly decreased CNV development in the laser-induced mouse model compared with controls, subsequently reducing the surface invaded by the new vessels. A proteomic approach revealed that RSG affects mainly protein involved in permeability or vascularization in the retina of mice, suggesting that a nutraceutical such as RSG could delay the progression of the appearance of AMD. These results reinforce previous studies showing that RSG can reduce hydroquinone-mediated production of reactive oxygen species and inflammasome activation and can promote autophagy during impaired protein clearance in ARPE-19 cells [9,10,11]. It was also shown to affect the VEGF production pathway.

Furthermore, a preliminary HPLC analysis performed after 21 days of RSG supplementation in mice showed that RSV aglycons, but mainly RSV metabolites (especially RSV-3-O-sulfate, RSV-4′-O glucuronide, and 3-O-glucuronide), were found in the retina and in the posterior pole. This is an important element considering that a clinical trial in colorectal cancer patients showed that oral absorption of RSV leads to an accumulation of these metabolites in the colon and rectum [16]. In a clinical study, Wang et al. measured RSV and its main metabolites in human eyes in patients with rhegmatogenous retinal detachment that were orally supplemented with a formulation containing 100 mg of RSV (one capsule daily for a total of three doses prior to surgical tissue resection) [27]. They showed that RSV and its metabolites were detectable in conjunctiva, aqueous humor, and vitreous humor (i.e., 17.19 ± 15.32 nmol/g RSV in conjunctiva and 62.95 ± 41.97 nmol/L RSV-3-O-sulfate in aqueous humor). These findings should be considered in further investigations, since colorectal cancer models indicate that these three metabolites exert an antitumor effect with a synergic action when present together [18], and they were also shown to have an effect in myopathy models [17]. Additional experiments are now needed to answer important questions posed by the results of this study, including the pharmacokinetics and bioavailability of RSG and the role of these metabolites in the action of RSG. More data are also required to define whether nutritional supplementation with RSG could increase the efficacy of anti-VEGF treatments as a therapeutic adjuvant or whether it could be used for secondary prevention to protect the second eye, which is a major issue. Such studies should be able to shed light on the interest of these compounds in the establishment of new therapeutic strategies to counter the progression of AMD.

## 4. Materials and Methods

### 4.1. Products Used for Animal Experimentation

Resvega^®^ (Laboratoires Théa, Clermont-Ferrand, France) is a nutraceutical composed of vitamin C 240 mg; E 30 mg; zinc 12.5 mg; Cu 1 mg; EPA 380 mg; DHA 190 mg; lutein 10 mg; zeaxanthin 2 mg; *trans*-resveratrol 30 mg. Nutrof^®^ (NUT) (Laboratoires Théa, Clermont-Ferrand, France) is a nutraceutical composed of vitamin C 60 mg; B3 18 mg; B6 2 mg; B2 1.6 mg; B1 1.4 mg; B9 200 µg; B12 1 µg; E 10 mg; zinc 7.5 mg; Cu 1 mg; Mg 1 mg; Se 25 µg; EPA 160 mg; DHA 80 mg; lutein 10 mg; zeaxanthin 2 mg; and resveratrol, RSV (Sigma-Aldrich, St. Quentin Fallavier, France).

### 4.2. Animal Studies

Animal use and care were approved by the Burgundy University Animal Experiments Ethics Committee, in accordance with the Federation of Laboratory Animal Science Associations (FELASA) (authorization number 2019100110475690_V2#22222). Only females 7 weeks of age were used for the experiments. Female C57BL/6 were purchased from Charles River Laboratories (Saint-Germain sur l’Arbresle, France). The animals were separated into 4 supplementation groups: Resvega^®^ (RSG), Nutrof^®^ (NUT), resveratrol (RSV), and a control group (Co). Mice were individually maintained on normal chow (control mice group) or fed per os with RSG (12 µM), NUT (12 µM), or with RSV (20 µM) for 14 days prior to the laser impacts. Prior to CNV induction, a drop of tropicamide 1% (Mydriaticum, Théa) was instilled on one eye of each mouse. Four argon laser impacts (300 Mw, diameter 75 µm for 50 ms, Vitra; Quantel Medical) were delivered on the fundus around the optic nerve using a slit lamp delivery system and a glass coverslip as a contact lens according to previously published procedures [28,29]. The validation of the injury was ascertained at the time of the laser shot by the appearance of a bubble. At days 14 and 21 after the laser impacts, the animals were subjected to retinal angiographies according to procedures previously published by our team [29,30]. Animals were anesthetized by subcutaneous injection of ketamine (100 mg/kg, Rompun 2%, Imalgène 100; Merial, Lyon, France) and xylazine (10 mg/kg, Rompun 2%; Bayer, Puteau, France). Their pupils were dilated using tropicamide 1% (Mydriaticum, Théa). Then the animals were placed in front of the camera of a confocal scanning laser ophthalmoscope (cSLO, Heidelberg Retina Angiograph I, Heidelberg Engineering, Heidelberg, Germany). After the acquisition of native fundus pictures using the 830 nm infrared laser of the cSLO, animals received a subcutaneous injection of fluorescein (75 mg/kg body weight, flurorescein-Na, Sigma-Aldrich, Saint Quentin Fallavier, France) and indocyanine green (ICG, 50 mg/kg body weight, Infracyanine, Serb, Paris, France). Single pictures and depth scan movies were taken at 10 min after dye administration. Photographs of the retinal and the choroidal vasculature were recorded at 488 nm for retinal vessel fluorescein angiography and at 795 nm for choroidal ICG angiography. Barrier filters at 500 and 810 nm provided the optimal cutoff at the respective peak fluorescence emission values for the two types of angiographies. The size of the square scan field was set at 20 °C. CNV was semi-quantified on fluorescein and ICG angiographies using ImageJ software. First, the optic nerve head surface was evaluated in pixels on native infrared images. Then the CNV areas were outlined on fluorescein and ICG angiography pictures that were taken at the same position. The ratio of the fluorescence of each laser impact to the optic nerve head area was calculated and averaged per eye (n = 4 impacts per eye, n = 9–10 eyes per group).

### 4.3. Proteomic Analysis of Retina

Mice retinas were lysed in 70 µL of SDS 1% in 50 mM triethylammonium bicarbonate buffer (TEAB), boiled for 10 min at 95 °C, and sonicated for 10 min (10 cycles of 30 s ON/30 s OFF, Bioruptor Pico, Diagenode). After clarification, protein concentration was determined by DC Protein Assay (Bio-Rad). Protein digestion was performed with S-Trap^TM^ micro spin column (Protifi, Huntington NY, USA) on 50 µg of lysates according to manufacturer’s instructions. Briefly, samples were reduced with 20 mM TCEP and alkylated with 50 mM CAA (chloracetamide) for 15 min at room temperature. Aqueous phosphoric acid was then added to a final concentration of 1.2% followed by the addition of S-Trap binding buffer (90% aqueous methanol, 100 mM TEAB, pH7.1). Mixtures were then loaded on S-Trap columns. Three extra washing steps were performed for thorough SDS elimination. Samples were digested with 2.5 µg of trypsin (Promega) at 47 °C for 2 h. After elution, peptides were vacuum dried and resuspended in 100 µL of 10% ACN and 0.1% TFA in HPLC-grade water prior to MS analysis.

### 4.4. NanoLC-MS/MS Protein Identification and Quantification

For each run, 1 µL (retina samples) was injected in a nanoRSLC-Q Exactive PLUS (RSLC Ultimate 3000) (Thermo Scientific, Illkirch-Graffenstaden, France). Peptides were loaded onto a µ-precolumn (Acclaim PepMap 100 C18, cartridge, 300 µm i.d.× 5 mm, 5 µm) (Thermo Scientific) and were separated on a 50 cm reversed-phase liquid chromatographic column (0.075 mm ID, Acclaim PepMap 100, C18, 2 µm) (Thermo Scientific). Chromatography solvents were (A) 0.1% formic acid in water and (B) 80% acetonitrile, 0.08% formic acid. Peptides were eluted from the column with the following gradient: 5% to 40% B (120 min), 40% to 80% (1 min). At 121 min, the gradient stayed at 80% for 5 min and, at 126 min, it returned to 5% to re-equilibrate the column for 20 min before the next injection. One blank was run between each replicate to prevent sample carryover. Peptides eluting from the column were analyzed by data dependent MS/MS, using the top-10 acquisition method. Peptides were fragmented using higher-energy collisional dissociation (HCD). Briefly, the instrument settings were as follows: resolution was set to 70,000 for MS scans and 17,500 for the data dependent MS/MS scans in order to increase speed. The MS AGC target was set to 3.106 counts, with maximum injection time set to 60 ms, while the MS/MS AGC target was set to 1.105, with maximum injection time set to 60 ms. The MS scan range was from 400 to 2000 *m*/*z*. Three separate mass spectrometry runs (i.e., technical replicates) were acquired for each biological replicate under the identical mass spectrometric conditions to account for instrument-related variability and to improve accuracy of the label-free quantification.

The MS files were processed with MaxQuant software version 1.6.14.0 and searched with Andromeda search engine against the UniProtKB/Swiss-Prot Homo sapiens or Mus musculus database (release April 2020, 20,365 entries). To search for parent mass and fragment ions, we set the mass deviation at 4.5 ppm and 20 ppm, respectively. The minimum peptide length was set to seven amino acids and strict specificity for trypsin cleavage was required, allowing up to two missed cleavage sites. Match between runs was allowed. Carbamidomethylation (Cys) was set as fixed modification, whereas oxidation (Met) and protein N-terminal acetylation were set as variable modifications. The false discovery rates (FDRs) at the protein and peptide level were set to 1%. Scores were calculated in MaxQuant, as described previously (PMID: 19029910). The reverse and common contaminants hits were removed from MaxQuant output. Proteins were quantified according to the MaxQuant label-free algorithm using LFQ intensities; protein quantification was obtained using at least 1 peptide per protein. Matching between runs was allowed.

### 4.5. MS Data Processing and Bioinformatics Analysis

Statistical and bioinformatics analyses, including heatmaps, were performed with Perseus software (version 1.6.12.0), available for free at www.perseus-framework.org (accessed on 2 October 2021). For the mice retina samples, we set four groups for statistical comparison, each containing up to 10 biological replicates. We then filtered the data to keep only proteins with at least 7 valid values in at least one group. Next, the data were imputed to fill missing data points by creating a Gaussian distribution of random numbers with a standard deviation of 33% relative to the standard deviation of the measured values and 1.8 standard deviation downshift of the mean to simulate the distribution of low signal values. We performed an ANOVA test, FDR < 0.05, S0 = 1. Hierarchical clustering of proteins that survived the test was performed in Perseus on logarithmized LFQ intensities after z-score normalization of the data, using Euclidean distances. After obtaining the ANOVA results, we next applied *t*-tests with Welch correction to identify significantly up- and down-regulated proteins in the RSG, NUT, and RSV groups compared with the control group (Co).

### 4.6. Functional Enrichment and Bioinformatics Analysis

Identification of enriched pathways and protein network analysis were performed with the Metascape online database [31] (http://metascape.org, (accessed on 7 July 2021)). Gene Ontology (GO) terms for the biological process, cellular component, and molecular function categories, as well as Kyoto Encyclopedia of Genes and Genomes (KEGG) pathways, Reactome Gene Sets, and CORUM were enriched. Accumulative hypergeometric *p*-values and enrichment factors were calculated and used for filtering. Remaining significant terms (*p*-value < 0.01, enriched genes ≥ 3) were then clustered onto a tree based on kappa—statistical similarities among their gene memberships. The most statistically significant term within a cluster was chosen as the one representing the cluster. A subset of enriched terms was selected and rendered as a network plot to further determine the relationship among terms, where terms with a similarity of >0.3 were connected by edges. Each term was represented by a circle node, where its size was proportional to the number of input proteins falling into that term, and its color represented its cluster identity. The network was visualized with Cytoscape (v3.1.2) with “force-directed” layout and with bundled edges.

### 4.7. Quantitative Analysis of Resveratrol and Metabolites by HPLC

For analysis of RSV metabolites, the presence of RSV aglycon and RSV metabolites was analyzed in retinas and posterior poles by LC-MS (Agilent Technologies, Les Ulis, France), as previously described [17,32].

### 4.8. Statistical Significance

Unless indicated in the legends of figures, the reported values represent the means of triplicates from one representative experiment repeated three times +/− SD. Statistical significance was determined using Mann–Whitney test at * *p* < 0.05, ** *p* < 0.01, or *** *p* < 0.001.

## 5. Conclusions

In a mice model of laser-induced choroidal neovascularization, we showed that a nutraceutical formulation enriched in omega-3 fatty acids and resveratrol, a naturally occurring polyphenol, could limit the extension of ocular neovascularization by up-regulating signaling pathways controlling angiogenic processes in the retina. These effects could be related to the capacity of resveratrol and its metabolites to accumulate in retinal tissue. Hence, our data highlight that Resvega^®^ supplementation might help to slow down the progression of AMD in patients and/or to potentiate anti-VEGF therapies. Nevertheless, further studies are required to fully decipher the underlying molecular mechanisms. It would also be of great importance to test whether the administration of the formulation after laser-induced retinal lesions in mice or in patients with already established AMD could also be effective in limiting ocular neovascularization and associated loss of vision. Such preclinical studies, which will be a next step, would definitely help in determining the preventive and/or curative potential of the formulation.

## Data Availability

The authors declare that all the data supporting the findings of this study are available within the article or from the corresponding authors upon reasonable request.
